# Hot Corrosion and Mechanical Performance of Repaired Inconel 718 Components via Laser Additive Manufacturing

**DOI:** 10.3390/ma13092128

**Published:** 2020-05-04

**Authors:** Qunli Zhang, Jie Zhang, Yifan Zhuang, Jinzhong Lu, Jianhua Yao

**Affiliations:** 1 Institute of Laser Advanced Manufacturing, Zhejiang University of Technology, Hangzhou 310023, China; zql@zjut.edu.cn (Q.Z.); zhangjie-zjut@foxmail.com (J.Z.); zhuangyifan56@163.com (Y.Z.); 2 Collaborative Innovation Center of High-end Laser Manufacturing Equipment, Zhejiang University of Technology, Hangzhou 310023, China; 3 School of Mechanical Engineering, Jiangsu University, Zhenjiang 212000, China; blueesky2005@163.com

**Keywords:** Inconel 718, hot corrosion, microstructural change, mechanical properties, laser additive manufacturing

## Abstract

Hot corrosion is one of the crucial failure modes of Ni-based superalloy components operating at high temperatures, which inevitably affects the subsequent mechanical properties of the alloys. In this research, damaged Inconel 718 alloy components with a pre-made trapezoid groove are repaired using laser additive manufacturing technique, and the change mechanisms of the microstructure and tensile properties of the repaired Inconel 718 alloy due to the hot corrosion in the salt mixture of 87.5 wt.% Na_2_SO_4_ + 5 wt.% NaCl + 7.5 wt.% NaNO_3_ at 650 °C for different durations are investigated. The results show that oxidation and Cr-depletion occur on the repaired components due to the hot corrosion, and the corrosion products are mainly composed of Cr_2_O_3_, Fe_3_O_4_, and Ni_3_S_2_. The tensile strength and elongation of the as-repaired specimens are 736.6 MPa and 12.5%, respectively. After being hot corroded for 50 h, the tensile strength increases to 1022.9 MPa and elongation decreases to 1.7%. However, after being hot corroded for 150 h, both tensile strength and elongation of the repaired specimens drop to 955.8 MPa and 1.2%, respectively. The mechanical performance alteration is highly related to thermal effects instead of the molten salt attack.

## 1. Introduction

Owing to the excellent strength and corrosion resistance at higher temperatures, nickel-based superalloys are widely used as the materials for the hot components in aero-engines and land-based gas turbines, such as blades, turbine discs, and combustion chambers [1,2,3]. However, due to the existence of various impurities such as sulfur, sodium, potassium, and vanadium in the fuels, SO_2_ and SO_3_ gases will be generated during the combustion process at high temperatures, which then react with oxygen and NaCl to form a layer of molten Na_2_SO_4_ on the surfaces of the hot components, thereby accelerating hot corrosion attack and the degradation of the alloys [4,5,6]. At high temperatures, the sulfur in the sulfate penetrates the oxide film and diffuses into the alloy to make it porous. At the same time, the reaction between the sulfate and the alloy makes the chemical composition of the molten salt complex and more corrosive, which further expedites the failure of the part [7,8].

Inconel 718 is one of the most popular nickel-based superalloys, which is designed for operation at temperatures up to 650 °C [9,10,11,12]. Because of the severe working environment, Inconel 718 components usually fail due to the occurrence of erosion, wear, corrosion, and fracture in long-term service conditions [2,13,14,15]. Based on the concept of green, economic, and environmental protection, these failed components can be repaired by laser additive remanufacturing techniques [16,17,18,19]. Onuike et al. [20] repaired the Inconel 718 parts with four different scan patterns and studied the wear properties of the repaired samples in different heat treatment conditions. They found that laser repair strategies are important to ensure high-quality remanufacturing of metal components. With regard to laser remanufactured parts, hot corrosion resistance should be seriously considered. According to previous researches [20,21,22,23], there is no macro-segregation in the laser-repaired region due to rapid melting and solidification in the laser deposition process. Furthermore, the microstructure of the repaired layer processed via laser additive manufacturing is different from that prepared via conventional methods such as forging and casting, which will inevitably result in difference of hot corrosion resistance for the repaired parts. Up to date, there are few studies focusing on high-temperature oxidation and hot corrosion of the nickel-based superalloys fabricated via laser remanufacturing and additive manufacturing techniques. Jia et al. [24] studied the high-temperature oxidation behavior of SLM-Inconel 718 alloy prepared with different laser energy densities in static air. The results showed that different laser energy densities were related to the density of the prepared material. The pore structure formed during laser selective melting can provide a path for oxygen during high-temperature oxidation and accelerate oxidation of the exposed Inconel 718 alloy. Sadeghi et al. [25] fabricated Inconel 718 alloy using an electron beam powder bed fusion technique (EB-PBF) and investigated the effect of different post treatments on the microstructure and oxidation behavior. They found that Cr depletion and increased oxygen diffusion in the EB-PBF fabricated specimen were affected by the coarse grains and formation of vacancies or voids along the grain orientation. Kang et al. [26] studied the effect of solution aging and hot isostatic pressing (HIP) on the microstructure and high-temperature oxidation (900, 1000 °C) behavior of Inconel 718 alloy fabricated via the selective laser melting (SLM) method. The results show that, after HIP treatment, the density of sample becomes higher and microstructural differences at the grain boundaries happen, resulting in the improvement of the oxidation resistance of the sample. Generally speaking, the alloys prepared via SLM or EB-PBF methods are different from those prepared via laser deposition technique, and the mechanism of hot corrosion is more complex than that of high-temperature oxidation. As for the effects of hot corrosion on the mechanical properties of superalloys, Mahobia et al. [27] studied the tensile behavior of pre-hot corrosion-treated Inconel 718 samples at temperatures of 550 and 650 °C and found that the tensile properties decreased slightly after long-term exposure due to strengthening precipitates instead of salt coating. They also revealed the effects of molten salt on the low-cycle behavior of Inconel 718 at 550 °C and found that the reduction of fatigue life was associated with early cracks resulting from the corrosion pits [28]. Liu et al. [29] investigated the hot corrosion behavior and the fracture strength degradation of Ti_3_SiC_2_ exposed to a mixed salt. The results showed that the fracture strength and weight loss of the hot corroded specimens decreased by 25% and 10%, respectively. Pradhan et al. [30] studied the high cycle fatigue behavior of pre-hot-corroded Inconel 718 in air at 600 °C and found that the fatigue life of the Inconel specimen decreased significantly due to the formation of pits on the surface caused by evaporation of chlorides. Moreover, the repaired parts also inevitably suffered serious corrosion when they operated in hot corrosion environment, which not only reduced the load-bearing performance of the parts, but also caused severe hot corrosion of grain boundaries, thus leading to significant alteration in mechanical properties. To the authors’ knowledge, there are few studies on the hot oxidation and mechanical performance of laser-repaired Inconel 718 parts for different exposure durations in the literature. Consequently, it is necessary to further investigate the hot corrosion behavior and mechanical performance alteration of laser-repaired Inconel 718 parts used in marine gas turbine due to hot corrosion, so as to establish the foundation for their extensive application.

In this research, the salt mixture of 87.5 wt.% Na_2_SO_4_ + 5 wt.% NaCl + 7.5 wt.% NaNO_3_ was selected as a corrosive medium at elevated temperature to simulate the operation environment of marine gas turbine [31]. Therefore, the hot corrosion behavior of laser-repaired Inconel 718 alloy in a marine-like environment and the associated microstructural evolution were studied. Meanwhile, the influence of hot corrosion on the tensile properties of laser-repaired Inconel 718 alloy was also investigated. These research results will provide the basis and reference for the long-term application of this alloy in harsh environments.

## 2. Materials and Methods

### 2.1. Materials and Laser Repair Experiment

**Figure 1:**
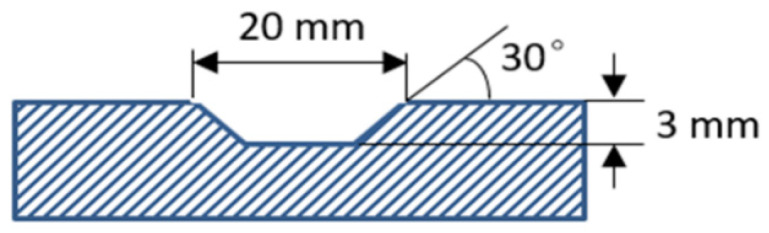
Schematic diagram of a trapezoid groove pre-made on the Inconel 718 substrate.

Firstly, a trapezoid groove with 3 mm depth, 20 mm top width, and 30° trapezoidal angle was pre-made on the hot-rolled Inconel 718 substrate using a wire cutting machine, as shown in Figure 1. The Inconel 718 substrate specimens were polished with sandpapers and cleaned with alcohol to remove oxide scales and greasy dirt. The Inconel 718 powder (53.5 Ni, 19 Cr, 18 Fe, 5 Nb, 3 Mo, 1 Ti, and 0.5 Al in wt.%) in the particle size range of 53–150 μm was used as the repair material. Before the repair process, the Inconel 718 powder was dried at 120 °C for 2 h in a vacuum furnace to remove moisture on the particle surface.

A fiber-coupled diode laser system (Laserline LDF400-2000, Mülheim-Kärlich, Germany) was used in the laser additive manufacturing process, and its main technical parameters were as follows: wavelength of 980 nm (continuous wave, CW) and maximum laser power of 2 kW. The output laser spot was circular with a diameter of 4 mm, and its energy density was uniformly distributed [32]. A self-made boiling type powder feeding device was used to deliver the powder particles into the molten pool.

The optimal processing parameters for the laser repair of damaged Inconel 718 alloy were obtained from preliminary experiments as follows [33]: laser power (*P*) of 900 W, scanning velocity (*V*) of 10 mm/s, powder-feeding rate (*F*) of 10 g/min, carrier gas flow rate of 15 L/h, and overlapping ratio (*R*) of 40%. The uplift of each layer is approximately equal to the thickness of the single cladded layer.

### 2.2. Hot Corrosion Testing

**Figure 2:**
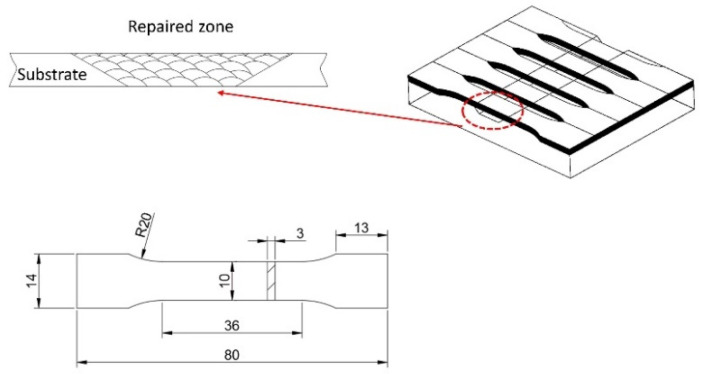
Drawing of distribution of laser-repaired zone in the tensile specimen and its dimensions.

The mechanical performance of the laser-repaired Inconel 718 alloy part was investigated after hot corrosion test in a salt mixture at 650 °C for different exposure times. Figure 2 shows the dimension of the tensile test specimen and the distribution of laser-cladded material in the tensile test specimen. It can be seen that the repaired area is located in the middle of the tensile specimen, and the laser-cladded material accounts for about 15.7% of the volume of the tensile specimen. All the test specimens were cleaned by acetone before hot corrosion testing. The salt mixture (corrosive medium) consisted of 87.5 wt.% Na_2_SO_4_ + 5 wt.% NaCl + 7.5 wt.% NaVO_3_, which simulated the marine-like environment [34]. The tensile test specimens were placed in rectangular alumina crucibles and immersed completely in the corrosive salt mixture at 650 °C in a muffle furnace (Shanghai Daheng Optics and Fine Mechanics Co., Ltd., Shanghai, China). To investigate the hot corrosion behavior and mechanical performance variation with exposure time, the specimens were hot-corrosion-tested for 50, 100 and 150 h, respectively. Each group had three parallel samples. After all the crucibles were completely cooled in air, the specimens were washed with boiled deionized water to remove residual corrosive medium on the surfaces and then dried in the oven at 150 °C.

### 2.3. Performance Characterization

The surface morphology and composition of the oxide scale on the specimen surface after the hot corrosion test were studied using a scanning electron microscope (SEM) (Carl Zeiss AG, Oberkochen, Germany) equipped with an energy-dispersive spectroscope (EDS) (Bruker AXS GmbH, Karlsruhe, Germany). The microstructural evolution of the corroded Inconel 718 specimens with different hot corrosion durations was also investigated. The metallographic samples prepared with the standard method were etched with the corrosive solution of CuCl_2_ (5 g) + C_2_H_5_OH (100 mL) + HCl (100 mL) for microstructure observation. The Vickers micro-hardness of the repaired Inconel 718 specimen after exposure to the molten salt for different corrosion times at 650 °C was evaluated using a digital micro-hardness tester (Shimadzu, Kyoto, Japan). The micro-hardness values were the average of 10 measurements for each specimen under the indentation load of 300 g for 10 s holding. The tensile tests of the repaired Inconel 718 samples after different hot corrosion times were carried out on an MTS SANS CMT5150 universal testing machine (MTS Systems Co., LTD, Shenzhen, China) at room temperature. For micro-hardness and tensile tests, three samples in same condition were used, and the average values of micro-hardness, tensile strength, and elongation were calculated to ensure the accuracy and reliability of the results.

## 3. Results

### 3.1. Microstructures and Phases

**Figure 3:**
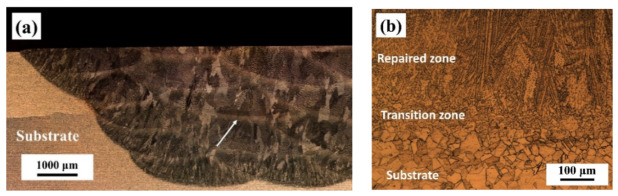
Cross-sectional microstructure of the repaired Inconel 718 alloy specimen: (**a**) at low magnification and (**b**) at high magnification.

Figure 3 shows the cross-sectional microstructure of the repaired Inconel 718 alloy specimen observed by optical microscopy. It can be seen that there were no obvious defects such as pores and cracks in the repaired zone. Since the trapezoid groove was filled with powder by laser irradiation layer by layer, the repaired zone appeared as an arc structure with multiple layers (Figure 3a), consisting of columnar dendrites which grow epitaxially along the deposition direction (Figure 3b) [35]. There was a transition zone between the repaired zone and the substrate, showing good metallurgical bonding characteristics [36].

**Figure 4:**
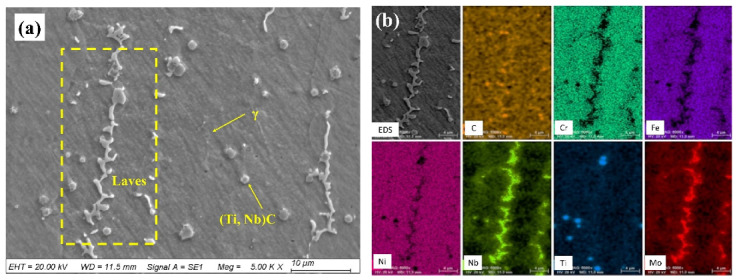
(**a**) Microstructure of the repaired Inconel 718 specimen and (**b**) energy-dispersive spectroscopy (EDS) mapping of the Laves phase.

The microstructures and elemental distributions of the repaired Inconel 718 specimens were further analyzed using SEM and EDS. As shown in Figure 4, there were a large number of white precipitates between the dendrites. The elemental distributions of the white precipitates and gray matrix phase in the repaired layer were analyzed by EDS mapping. The results show that the white precipitates were rich in Nb and Mo elements, but poor in Fe, Cr, and Ni elements, while the gray matrix phase was the opposite. Based on a report in the literature [35], the white precipitates are Laves phases and the gray matrix is the γ matrix. It is also clearly seen that many (Ti, Nb)C particles were precipitated in the γ matrix.

### 3.2. Hot Corrosion Performance

**Figure 5:**
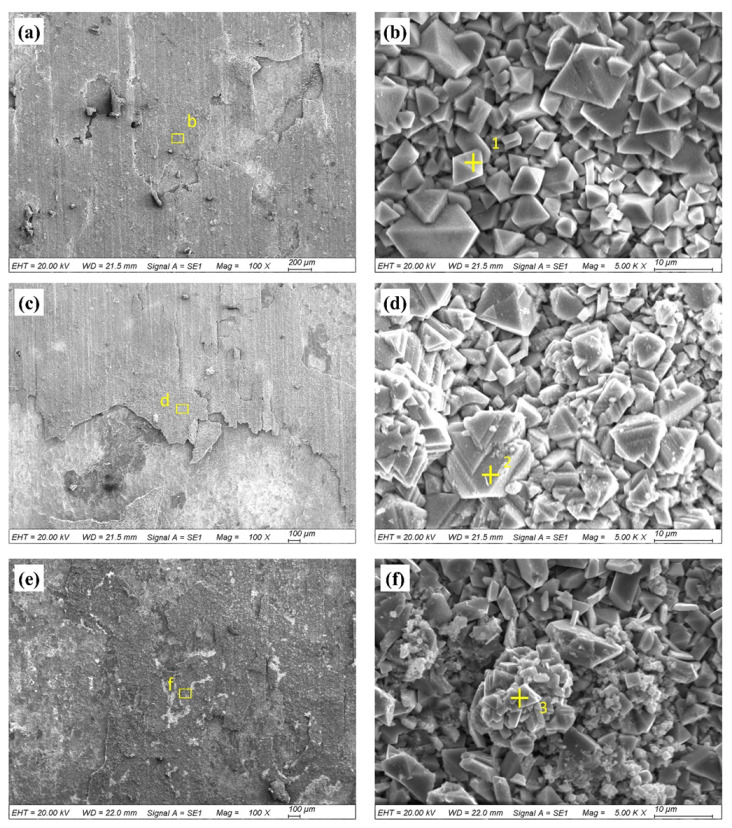
Surface morphologies of the repaired Inconel 718 specimen exposed to the molten salt at 650 °C for 50 h (**a**) at low magnification and (**b**) at high magnification, for 100 h (**c**) at low magnification and (**d**) at high magnification, and for 150 h (**e**) at low magnification and (**f**) at high magnification.

Figure 5 shows the surface morphologies of the repaired Inconel 718 specimens exposed to the molten salt at 650 °C for different time periods. It can be seen that the corrosion products gradually formed in the repaired zone during the hot corrosion process. Due to the attack of the molten corrosion salt, the oxide scales spalled off the surface and, thus, made the surface very rough and porous. In other words, the surface appearance deteriorated with the corrosion time increasing. The spines with different sizes were found in the corrosion scales. The EDS results from Table 1 reveal that these spines mainly consist of elements of O, Fe, Cr, and Ni.

**Figure 6:**
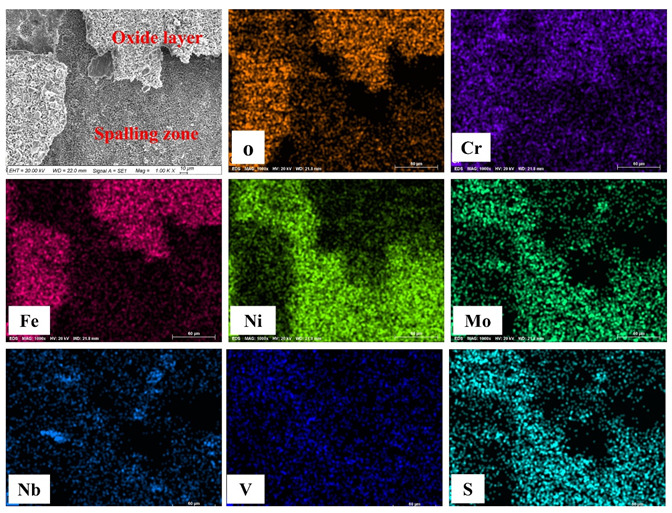
EDS mapping results of the hot corroded sample surface after 100 h test.

Figure 6 presents the EDS mapping results of the hot corroded sample surface after 100 h exposure. The surface had two distinct regions. One was the oxide layer, which was rich in O, Fe and Cr elements, which was identified as containing Fe_3_O_4_ and Cr_2_O_3_. The other was the spalling zone rich in Ni and S elements, indicating the formation of Ni_3_S_2_ during the hot corrosion. It is generally known that the formation of Fe and Cr oxides mainly depends on the outward diffusion behavior of Fe and Cr elements, and the oxide scale thickness usually increases with the corrosion proceeding [36,37].

**Figure 7:**
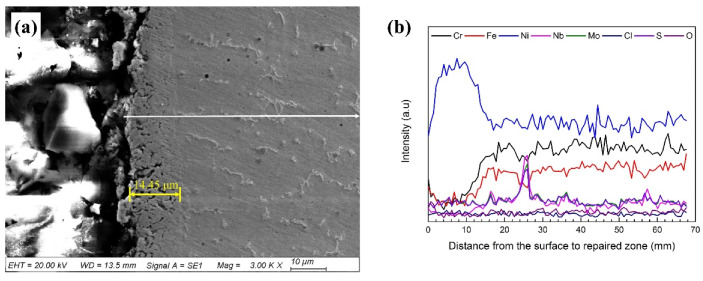

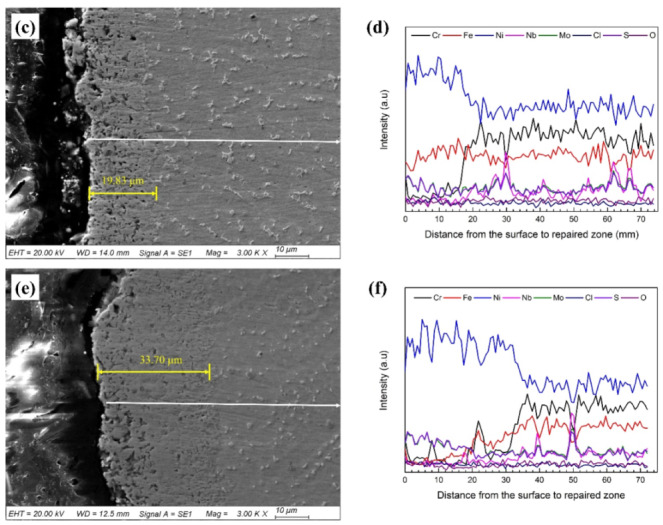
Cross-sectional SEM micrographs and corresponding linear analysis results of the repaired Inconel 718 specimen after the hot corrosion for 50 h (**a**) SEM image and (**b**) element distribution, for 100 h (**c**) SEM image and (**d**) element distribution, and for 150 h (**e**) SEM image and (**f**) element distribution.

Figure 7 shows the cross-sectional morphology and EDS line scanning results of the repaired Inconel 718 specimen corroded at 650 °C for different times. On all specimens, an Ni-rich region and pits were observed. In the Ni-rich region, the scale thickness increased with hot corrosion time, which was evaluated as 14.45 μm for 50 h, 19.83 μm for 100 h and 33.70 μm for 150 h. The EDS line scanning analyses of the Ni-rich region and the repaired Inconel 718 layer show that Cr and Fe had a lower peak in the Ni-rich region and then gradually increased toward the repaired layer. Combined with the EDS mapping results in Figure 5, the corrosion product in the Ni-rich region was Ni_3_S_2_, which was caused by the inward diffusion of S element during the hot corrosion. Furthermore, due to the attack of chlorine ions, the Ni-rich region had many pits which led to the pores.

### 3.3. Microstructural Change Due to Hot Corrosion

**Figure 8:**
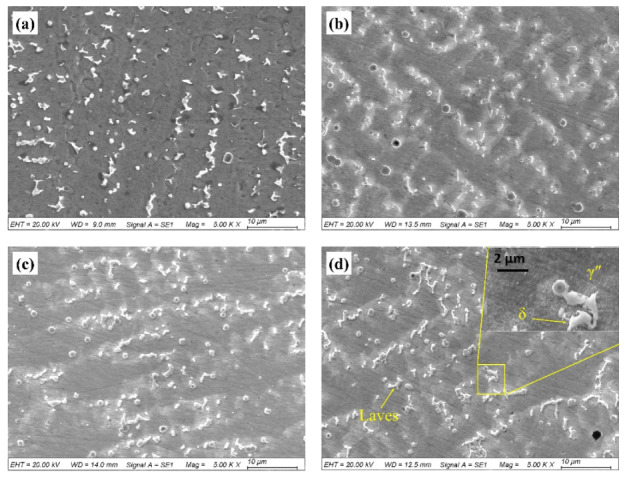
Microstructure of the repaired Inconel 718 alloy specimen exposed to the molten salt at 650 °C for (**a**) 0 h, (**b**) 50 h, (**c**) 100 h, and (**d**) 150 h.

Figure 8 shows the microstructures of the repaired Inconel 718 alloy specimens exposed to the molten salt at 650 °C for different time duration. It was found that, with the increase in the exposure time, the γ” phase precipitated around the residual Laves phase and gradually grew up [11]. When the test time was over 150 h, the δ phase began to precipitate around the residual Laves phase. In general, the γ′ and γ” phases precipitated from the supersaturated solid solution. According to a previous study [38], the γ′ phase usually precipitates at the temperature ranging from 593 to 816 °C and dissolves at the temperature between 843 and 871 °C. As for the γ” phase, the precipitation temperature is about 595–870 °C, but it usually forms at the temperature of 732–760 °C. The dissolution temperature of this phase is 870–930 °C. Therefore, with proceeding of the hot corrosion testing, the strengthening phases precipitate around the residual Laves phase. However, since the γ” phase is a metastable intermetallic phase, it usually changes to a δ phase when the temperature is over 650 °C or when exposed to the hot corrosive environment for a long time (Figure 8d) [22,39].

### 3.4. Mechanical Properties

#### 3.4.1. Micro-Hardness

**Figure 9:**
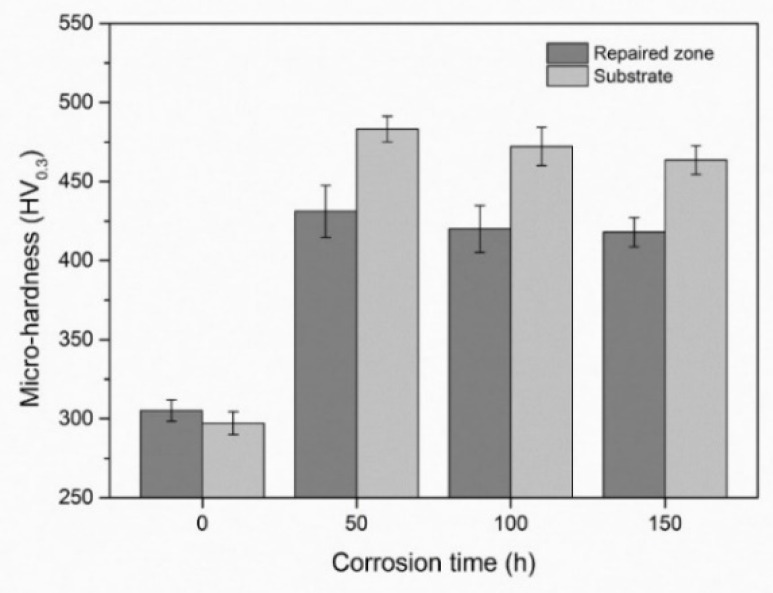
Micro-hardness variation of the repaired Inconel 718 alloy specimen with different exposure time to the molten salt at 650 °C.

Figure 9 shows the average micro-hardness values of the repaired zone and the substrate after different corrosion test times. It is evident that the hardnesses of the repaired zone and the substrate increased after the hot corrosion test; however, with the corrosion proceeding, the hardness slightly decreased for both the repaired zone and the substrate. This is because the strengthening phase and precipitation of the δ phase grew up with time in the corrosion test condition. Similar results were reported by Anderson showing that the transformation of the γ” phase to the δ phase resulted in dropping of the hardness of the Inconel 718 alloy at 875 °C [39].

#### 3.4.2. Tensile Strength

Table 2 shows the tensile properties of the laser-repaired Inconel 718 samples and the hot-rolled substrate. It can be seen that the tensile strength and elongation of the repaired Inconel 718 alloy specimens were lower than those of the substrate material, which were 736 MPa and 12.5%, respectively, only reaching 78.8% and 22% of the substrate. However, it should also be noted that the elongation of the laser-repaired sample was higher than the casting standard (5%) and wrought standard (12%) of the Inconel 718 alloy.

**Figure 10:**
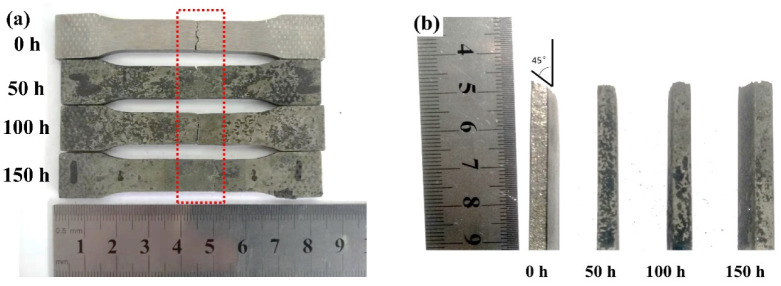
Photographs of the tensile-tested repaired Inconel 718 specimens with pre-hot corrosion testing in the molten salt at 650 °C: (**a**) failed specimens and (**b**) fracture surfaces.

Figure 10 photographically shows the repaired Inconel 718 alloy specimens after tensile testing at room temperature, which were pre-corrosion-tested in the molten salt at 650 °C for different exposure times. It can be seen that the fracture occurred in the repaired zone rather than in the interface zone for all specimens, which indicates that the repaired zone was well bonded with the substrate and the repaired zone was the weakest area. It should be noted that the fracture surface of the specimen without hot corrosion was at a 45° angle with the longitudinal axis of specimen (Figure 10b), where the maximum shear stress occurred, exhibiting ductile fracture characteristics [42]. However, after the hot corrosion testing, the specimens showed brittle fracture behavior, which was characterized by a flat fracture surface, that is, the surface was perpendicular to the longitudinal axis of specimen, where the maximum principal stress occurred (Figure 10b).

**Figure 11:**
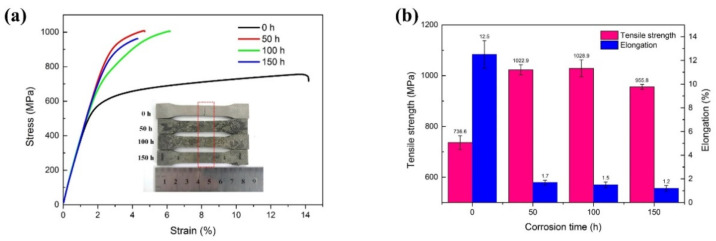
(**a**) The stress–strain curves and (**b**) mechanical properties of laser-repaired Inconel 718 alloy specimen exposed to the molten salt at 650 °C for different corrosion times.

Figure 11 presents the stress–strain curves and mechanical properties of the repaired Inconel 718 alloy specimens after exposure to the molten salt at 650 °C for different time lengths. It shows that the as-repaired specimen without hot corrosion had a lower tensile strength (736.6 MPa) but larger elongation (12.5%). For the hot-corroded specimens, the tensile strength was improved while the ductility was reduced. As shown in Figure 11, after 50 h hot corrosion, the tensile strength of the specimen increased to 1022.9 MPa and the elongation decreased to 1.7% due to precipitation of the γ” phase. However, after the 100 h hot corrosion test, the tensile strength and elongation of the specimen did not change very much, compared with the 50 h test condition. However, it should be noted that, after the 150 h corrosion test, the tensile strength and elongation of the repaired specimens decreased to 955.8 MPa and 1.2%, respectively. As discussed above, the molten salt made the specimen’s surface rough and defective, and it caused the formation of thicker oxide scales and a porous Ni-rich layer on the surface of the sample. A large number of cracks and pits were detected in the scales, which could result in premature failure and mechanical performance degradation of the repaired specimens. On the other hand, the tensile strengths of the specimens which were hot corroded for 50 h and 100 h were higher than those of the as-repaired specimens without hot corrosion, which was attributed to precipitation of the strengthening phases instead of the destruction of the molten corrosive salt. Similar results were reported by Mahobia et al. [43]. It is interesting to note that, over 150 h corrosion testing, the tensile strength and elongation of the specimens began to decrease significantly due to the precipitation of the δ phase around the Laves phase. Similar results were also reported by Kuo et al. [44], who found that the interdendritic δ phases were undesirable and had an adverse effect on the mechanical properties.

**Figure 12:**
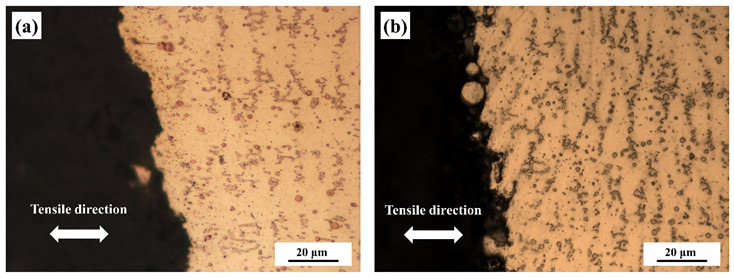

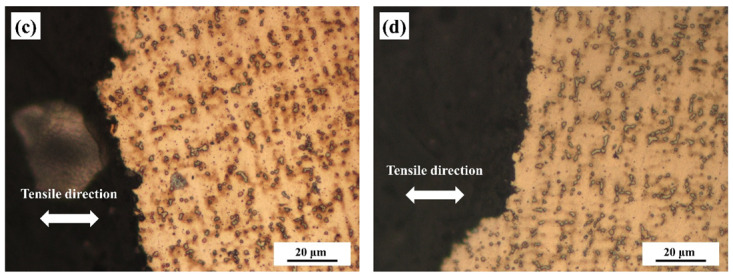
The cross-sectional microstructure near the fractured surface of the repaired Inconel 718 alloy specimen with pre-hot corrosion for different exposure times: (**a**) 0 h, (**b**) 50 h, (**c**) 100 h, and (**d**) 150 h.

Figure 12 shows the cross-sectional microstructure near the fractured surface of the repaired Inconel 718 alloy specimen with different hot corrosion times. It can be seen that the fracture mainly extended along the interface between the Laves phase and γ matrix. This is because the strengthening elements such as Nb, Mo, and Ti segregated in interdendrites to form the Laves phase during the laser repair process, resulting in lower tensile stress [21]. Moreover, with the increase of hot corrosion time, a lot of γ″ phase structures precipitated around the Laves phase, which enhanced the tensile strength of the specimens. However, when the hot corrosion time was over 150 h, some small δ phase structures precipitated around the Laves phase, resulting in depletion of the γ″ strengthening phase, which had an adverse effect on the strength of the repaired Inconel 718 specimen.

## 4. Discussion

The corrosion products of the laser-repaired Inconel 718 alloy specimens exposed to the molten salt (87.5 wt.% Na_2_SO_4_ + 5 wt.% NaCl + 7.5 wt.% NaVO_3_) for different time periods mainly consisted of Cr, Fe-rich oxides, and Ni sulfides. The continued formation of the Cr, Fe-rich oxides led to reduction of the oxygen partial pressure and an increase of the sulfur partial pressure at the interface between the molten salt and the alloy surface [33]. The formation of the oxide is related to the outward diffusion behavior of the Cr and Fe elements, which subsequently react with oxygen, as expressed in Equations (1) and (2).

2Cr + 3/2O_2_ = Cr_2_O_3_, (1)

3Fe + 2O_2_ = Fe_3_O_4_. (2)

As demonstrated in the elemental distribution analyses, the Cr-depletion and Ni-rich layer (Ni-rich region in Figure 7 is full of nickel sulfides governed by the inward diffusion of S elements). This is because Ni is more susceptible to be attacked by sulfur according to Equation (3).

(3) $$9Ni\;+\;{2SO}_{4}^{2-}\;=\;6NiO\;+\;{Ni}_{2}S_{2}\;+\;2O^{2-}$$

It should be noted that the formation of nickel sulfides can be accelerated due to the existence of cracks and pits caused by NaCl and thermal expansion. This is because NaCl can induce Cl_2_, which makes the oxide layer porous, thus providing a diffusion channel for S element, as expressed by Equation (4). Therefore, the hot corrosion behavior is related to sulfidation and oxidation [45].

8NaCl + 2Cr_2_O_3_ + 5O_2_ = 4Na_2_CrO_4_ + 4Cl_2_. (4)

It can be seen that the protective oxide layer is gradually dissolved and destroyed. Meanwhile, the thickness of the nickel sulfides increases along with hot corrosion time due to the continuous inward diffusion of S element. Therefore, the hot corrosion mechanism of the laser-repaired Inconel 718 alloy agrees with the sulfurization–oxidation model and alkali-base melting model. Generally speaking, the corrosive molten salts preferentially attack the grain boundary and phase interface. However, since the temperature of hot corrosion is lower than the recrystallization temperature of the Inconel 718 alloy, the grain size of laser-repaired Inconel 718 does not change significantly [11]. As for Nb-rich precipitates, Laves and (Ti, Nb)C are more prevalently attacked by molten salt [37]. With the hot corrosion time extending, Nb element diffuses into the γ matrix from the Laves phase and forms the strengthening phases, which effectively reduces the segregation of Nb and helps to improve the corrosion performance [46].

The micro-hardness and tensile testing results of pre-hot corrosion-tested specimens reveal that the mechanical performance alteration is highly related to thermal effects instead of the molten salt attack. This is because the as-repaired Inconel 718 lacks the strengthening phases of γ” and γ′, and it needs to be aged to obtain the precipitates [47]. During hot corrosion testing, the thermal effect promoted precipitation of the strengthening phase precipitates around the Laves phase, which certainly increased the hardness and tensile properties of the repaired Inconel 718 alloy specimen. However, with the hot corrosion time extending, the δ phase began to precipitate around the Laves phase by consuming the strengthening elements between the dendrites, which further aggravated the barrenness of interdendritic strengthening elements, decreased the strength of the matrix, and favored the initiation and propagation of cracks in the tensile process.

The mechanical properties and the hot corrosion properties of the laser-repaired Inconel 718 alloy are highly related to its microstructure. Qi et al. [48] found that the standard heat treatment can improve the distribution of elements, refine the grain size, and improve the mechanical properties of the Inconel 718 alloy. Asala et al. [46] also found that the homogenization of the composition and microstructure by standard heat treatment can effectively improve the hot corrosion performance of the ATI 718 Plus alloy. Therefore, the standard heat treatment before hot corrosion can be introduced to improve the hot corrosion and mechanical properties of the laser-repaired Inconel 718 parts.

## 5. Conclusions

The failed components of the Inconel 718 alloy with a pre-made trapezoid groove were repaired using a laser additive manufacturing technique. The alterations of microstructure and mechanical properties of the repaired Inconel 718 alloy specimen after exposure to the salt mixture of 87.5 wt.% Na_2_SO_4_ + 5 wt.% NaCl + 7.5 wt.% NaNO_3_ at 650 °C for different corrosion times were studied. The following conclusions can be drawn from this research:

(a) The pre-made trapezoidal groove was successfully repaired using laser additive manufacturing without obvious defects. The microstructure of the laser-repaired zone mainly consisted of the Laves phase, γ matrix, and (Ti, Nb)C.

(b) A corrosion oxide layer and Cr-depletion layer formed on the surface of specimens during the hot corrosion testing. The Cr-depletion and Ni-rich layer was identified as Ni_3_S_2_, which increased with the hot corrosion proceeding.

(c) With the increase in hot corrosion time, the strengthening phases of γ” and γ′ precipitated around the Laves phase, resulting in a significant increase in micro-hardness and tensile strength of the repaired Inconel 718 alloy. However, the δ phase began to precipitate around the Laves phase and resulted in a slight decrease in micro-hardness and tensile strength when the hot corrosion time was 150 h.

(d) The specimens pre-corrosion tested for different times were all fractured in the repair zone under tensile testing, where the fracture mainly extended along the interface between the Laves phase and γ matrix.

## Figures and Tables

**Table 1:** EDS analysis results of the areas indicated in Figure 5 (wt.%)

Area	O	Fe	Cr	Ni
1	32.85	42.52	1.79	27.50
2	32.33	34.56	10.71	22.40
3	29.32	33.26	13.31	24.11

**Table 2:** Tensile properties of the laser-repaired samples and the substrate.

Sample	Tensile Strength (MPa)	Elongation (%)
As-repaired specimen	736.6	12.5
As-received substrate	934.9	56.7
Casting standard (Q/5B 453-1995) [40]	825	5
Wrought standard (Q/3B 548-1996) [41]	1340	12
